# Fungal Biotechnology for Sustainable Biocomposites: From Mycelium Growth to Material Translation

**DOI:** 10.3390/molecules31183272

**Published:** 2026-09-15

**Authors:** The Hong Phong Nguyen, Lachlan Thompson, Mostafa Nikzad, Huseyin Sumer

**Affiliations:** 1 Department of Mechanical and Product Design Engineering, School of Engineering, Swinburne University of Technology, Hawthorn, VIC 3122, Australia; peternguyen@swin.edu.au (T.H.P.N.); lachlanthompson@swin.edu.au (L.T.); 2 Department of Chemistry and Biotechnology, School of Science, Computing and Emerging Technologies, Swinburne University of Technology, Hawthorn, VIC 3122, Australia

**Keywords:** mycelium-based biocomposites, fungal biotechnology, thermal insulation materials

## Abstract

Fungal biotechnology has emerged as a promising platform for the development of sustainable biocomposites, leveraging the intrinsic ability of fungi to transform complex polymeric substrates into structurally integrated materials. This review critically evaluates mycelium-based biocomposites (MBCs), with particular emphasis on the relationships between fungal biosynthesis, substrate transformation, processing strategies, and resulting material properties. Key biochemical components, including chitin, chitosan, and β-glucans, are examined in terms of their molecular structures, biosynthetic pathways, and contributions to composite performance. The chemical modification of polymeric substrates during fungal colonisation, including enzymatic degradation, substrate remodelling, and interfacial bonding mechanisms, is critically discussed. The review further examines chemical functionalisation, hybrid reinforcement, and densification strategies for tailoring mechanical performance, thermal insulation, fire resistance, and durability, highlighting recent advances in processing-driven material optimisation. Applications in packaging, construction, insulation, environmental remediation, and functional materials are critically reviewed alongside sustainability considerations, including biodegradability, circularity, and life-cycle impacts. Finally, current challenges and future research directions are discussed, emphasising the integration of synthetic biology, advanced materials chemistry, and digital bio-fabrication to enable scalable, high-performance, and multifunctional MBCs.

## 1. Introduction

The unprecedented growth in global consumption of materials for construction, packaging, transportation, and consumer products has been accompanied by an increasing reliance on petroleum-derived polymers and polymer composites, which currently dominate many industrial sectors due to their low cost, lightweight nature, processability, and durability despite being fundamentally dependent on finite fossil carbon resources [1,2,3]. The production of conventional synthetic polymers is intrinsically energy-intensive and contributes substantially to greenhouse gas emissions throughout their life cycle, creating significant challenges for achieving carbon neutrality and the broader goals of a circular bioeconomy [4,5,6]. Furthermore, the exceptional durability that makes synthetic polymers commercially attractive also results in their prolonged environmental persistence, causing the accumulation of plastic waste in terrestrial, freshwater, and marine ecosystems worldwide [1,7,8]. The fragmentation and weathering of plastic debris generate micro- and nano-plastics that are now recognised as ubiquitous environmental contaminants capable of dispersion through air, water, soils, and food chains, raising growing concerns regarding ecosystem health and potential human exposure [9,10,11]. Consequently, there is an increasing demand for alternative material platforms, particularly as global plastic production has exceeded 400 million tonnes annually and recycling rates remain below 10%, that combine reduced environmental impact with renewable feedstocks, low-energy manufacturing, and end-of-life biodegradability [12,13,14,15]. Similar sustainability-driven developments are also evident in the construction materials sector, where increasing attention has been directed towards the utilisation of recycled aggregates, recycled concrete powders, and carbon-mineralised materials to reduce resource consumption and greenhouse gas emissions [16,17,18,19,20]. Recent studies have demonstrated that recycled aggregate-derived mortars can achieve satisfactory mechanical performance while promoting waste valorisation and resource efficiency [17,18,19,20]. Likewise, CO_2_ mineralisation technologies have shown considerable potential for enhancing the performance of recycled cementitious materials while simultaneously contributing to carbon sequestration and circular economy objectives [16]. These developments highlight the broader transition towards sustainable materials engineering and provide important context for the emergence of biologically derived material platforms such as MBCs.

Among the emerging bio-based material technologies, fungal biotechnology has attracted considerable attention because filamentous fungi possess highly adaptable metabolic and enzymatic systems capable of modifying, restructuring, and valorising complex organic substrates into functional biomaterials [21,22,23]. Through the secretion of oxidative and hydrolytic enzymes, many fungal species can efficiently colonise lignocellulosic biomass and other heterogeneous carbon sources, converting agricultural, forestry, and industrial residues into consolidated material structures with minimal waste generation [23,24,25]. In contrast to conventional manufacturing routes that often require elevated temperatures, high pressures, and energy-intensive processing steps, fungal bio-fabrication relies primarily on biological growth under relatively mild environmental conditions, thereby reducing external energy demands and associated carbon emissions. However, the overall environmental performance of MBCs remains dependent on factors such as feedstock preparation, sterilisation, drying, densification, post-processing treatments, and chemical modification strategies, and should therefore be evaluated on a case-by-case basis using appropriate life-cycle assessment methodologies [21,26,27]. These characteristics position fungi as attractive biological tools within emerging circular manufacturing systems, where low-value biomass streams can be transformed into value-added products through biologically directed assembly processes [24,26,28].

A distinguishing feature of fungal bio-fabrication is the intrinsic ability of filamentous fungi to self-organise into three-dimensional networks of interconnected hyphae, which act simultaneously as biological growth structures, transport pathways, and natural binding agents within composite systems [22,24,29]. During substrate colonisation, the expanding mycelial network penetrates, surrounds, and mechanically interlocks with substrate particles while depositing extracellular polymers and cell-wall constituents that contribute to cohesion and structural integrity [24,25,28]. As a result, material formation emerges directly from biological growth rather than from the external assembly of chemically synthesised constituents, representing a fundamentally different manufacturing paradigm from traditional composite fabrication [21,22,26]. The architecture and density of the resulting hyphal network are strongly influenced by fungal species, nutrient availability, substrate composition, and cultivation conditions, leading to substantial variations in the morphology and performance of the final material [24,28,30].

Within this rapidly evolving field, mycelium-based biocomposites (MBCs) have emerged as one of the most extensively studied fungal materials due to their capacity to integrate renewable biomass feedstocks with fungal growth processes to generate lightweight, biodegradable, and potentially carbon-efficient materials [21,25,27]. Rather than functioning as simple bio-based substitutes for conventional materials, MBCs are increasingly recognised as biohybrid material systems in which the physicochemical characteristics of the substrate interact dynamically with fungal metabolism, enzymatic activity, and hyphal development during fabrication [24,28,31]. The composition of lignocellulosic feedstocks, including cellulose, hemicellulose, lignin content, nutrient availability, particle size, moisture content, and surface chemistry, directly affects fungal colonisation behaviour and subsequently influences composite microstructure and material properties [24,28,31]. Equally important, processing interventions such as substrate pretreatment, moulding approaches, growth control strategies, drying protocols, and post-processing operations can significantly alter interfacial bonding, density, porosity, and performance outcomes [27,28,31].

**Figure 1:**
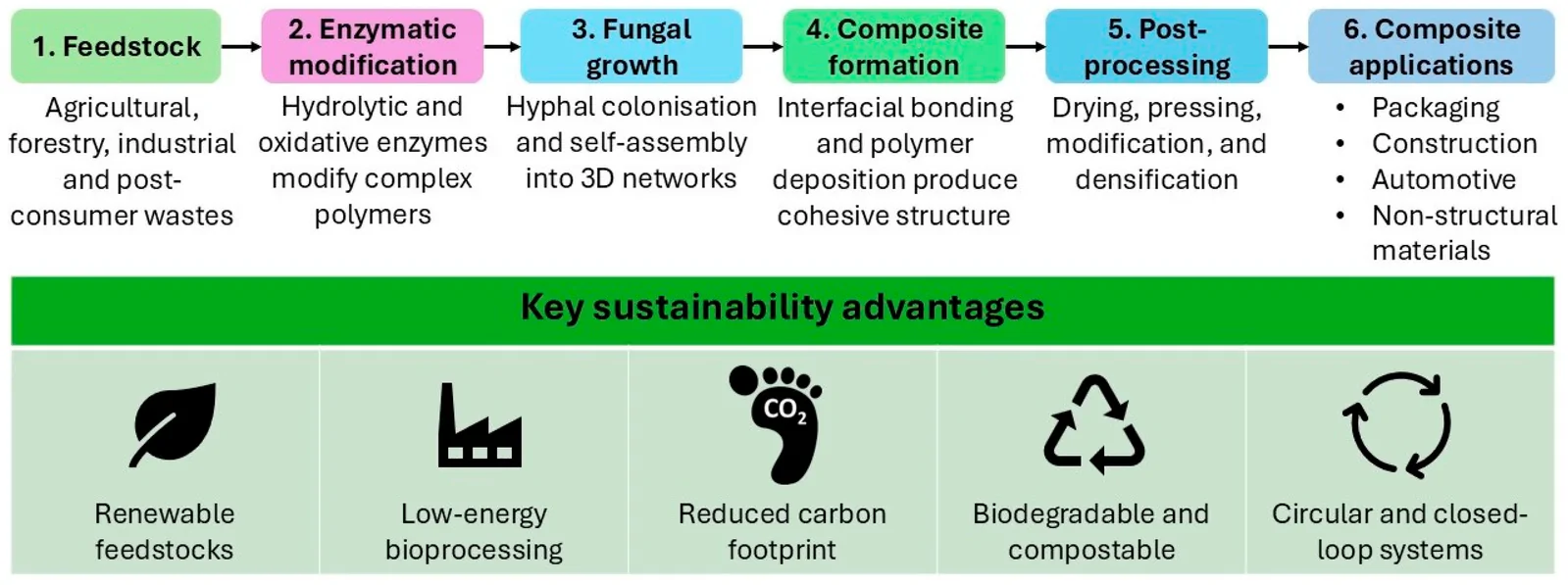
Fungal biotechnology roadmap for MBCs. Agricultural, forestry, industrial, and post-consumer feedstocks are transformed through fungal colonisation, enzymatic substrate degradation, hyphal network formation, composite consolidation, and post-processing operations to produce functional materials. Key sustainability benefits include renewable feedstocks, low-energy manufacturing, reduced carbon footprint, biodegradability, and compatibility with circular economy principles.

Despite substantial advances in the development of mycelium-based materials, many fundamental questions remain unanswered regarding the relationships between fungal biosynthesis, substrate transformation, hierarchical structure formation, and the resulting mechanical, thermal, acoustic, and moisture-related properties [24,27,30]. Establishing robust process–structure–property relationships is particularly important for advancing MBCs from laboratory-scale demonstrations toward predictable and scalable engineering materials suitable for industrial implementation [21,24,31]. Accordingly, this review examines MBCs from the perspective of biohybrid material systems, with particular emphasis on fungal biosynthesis mechanisms, substrate–fungus interactions, processing strategies, and the multiscale structure–property relationships that govern material performance and application potential. The conceptual framework adopted throughout this review, including fungal biosynthesis, substrate transformation, processing strategies, material optimisation, sustainability, and application development, is summarised in Figure 1.

Several recent reviews have examined MBCs from the perspectives of sustainability, construction applications, packaging materials, fabrication techniques, and commercial development [21,24,25,26,27,31]. However, comparatively less attention has been devoted to establishing integrated links between fungal biosynthesis, substrate transformation, chemical evolution, processing strategies, and material performance. The distinctive contribution of this review is its process–structure–property framework, which connects molecular-scale fungal biology and chemistry with macroscale composite behaviour. In addition to summarising current applications and sustainability considerations, this review critically evaluates how fungal cell-wall composition, enzymatic substrate modification, interfacial interactions, chemical functionalisation, and post-processing treatments collectively govern the engineering performance of MBCs. Through this integrated perspective, the review provides a comprehensive foundation for the future design, optimisation, and commercialisation of high-performance MBCs.

## 2. Fungal Biosynthesis and Molecular Composition

### 2.1. Chemical Composition of Mycelial Cell Walls

The fungal cell wall is a complex and dynamic composite structure that provides mechanical support, protects against environmental stress, and serves as the primary structural framework governing the performance of mycelium-based materials [32,33,34]. The cell wall is predominantly composed of polysaccharides, including chitin, chitosan, and β-glucans, which form an interconnected network that governs cell morphology, mechanical integrity, and interfacial interactions within fungal biomaterials [33,34]. The relative abundance and organisation of these cell-wall constituents can vary substantially among fungal species, developmental stages, and growth conditions. Species commonly employed in MBC production, including members of the genera *Pleurotus*, *Ganoderma*, and *Trametes*, may exhibit differences in the proportions of chitin, chitosan, β-glucans, proteins, and associated cell-wall components [32,33,34,35,36,37,38,39,40]. Such compositional variability can influence cell-wall architecture, moisture sorption behaviour, interfacial interactions, and the resulting mechanical and functional properties of MBCs. Consequently, fungal species selection represents an important design parameter in the development and optimisation of MBCs.

Chitin functions as the primary reinforcing phase of fungal cell walls due to its semi-crystalline microfibrillar architecture and high hydrogen-bond density [33,34,35]. The extensive intra- and intermolecular hydrogen bonding gives chitin high crystallinity and stiffness, making it an important contributor to the load-bearing capacity of fungal tissues and mycelium-derived materials [35,36,37]. Embedded within the chitin scaffold are β-glucans, predominantly β-(1 → 3)-glucans with β-(1 → 6)-linked branches, which form highly interconnected networks responsible for cell-wall cohesion and structural organisation [32,33,34].

**Figure 2:**
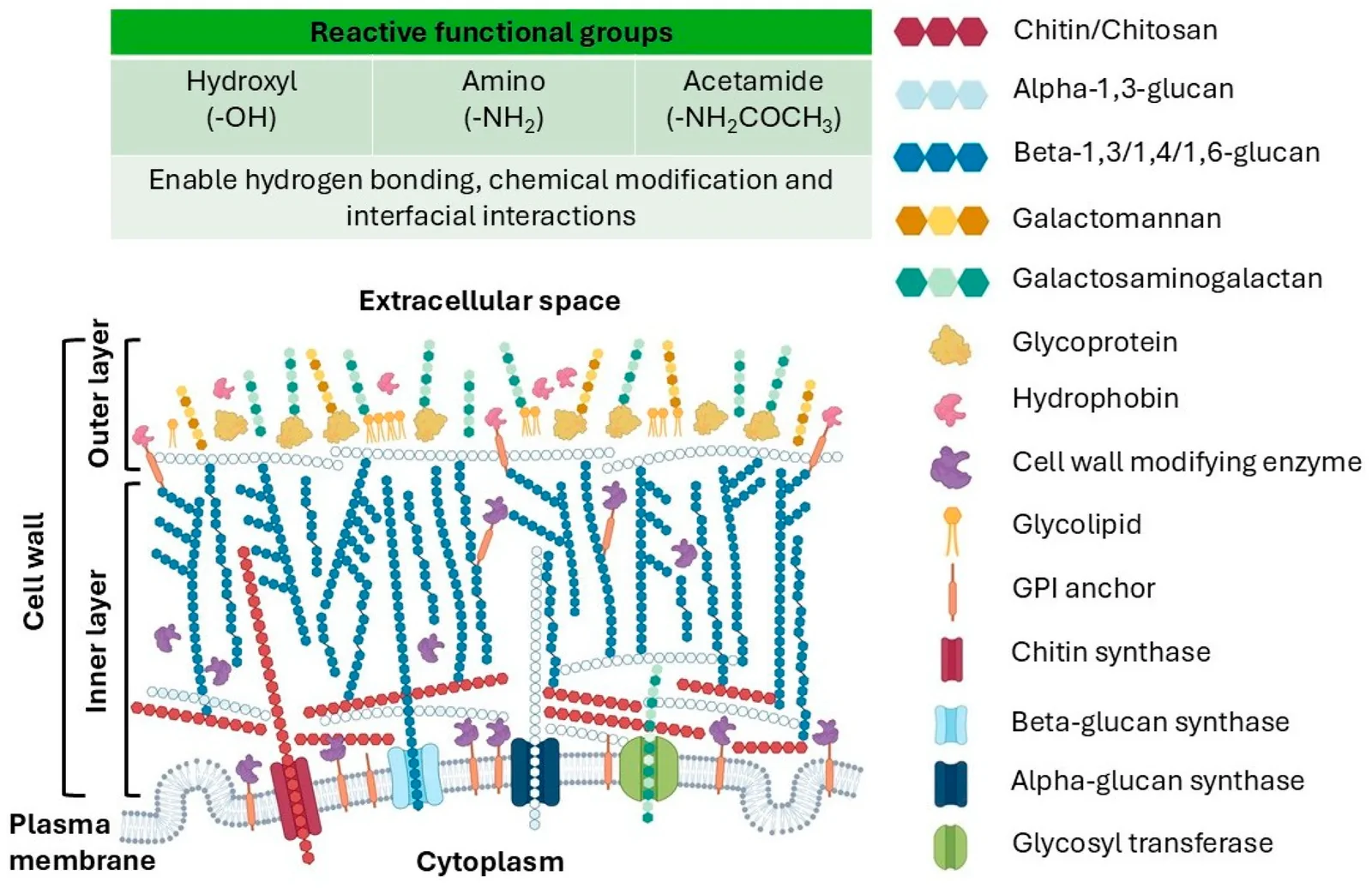
Schematic representation of fungal cell-wall architecture and molecular composition. Chitin microfibrils form the primary reinforcing framework, while chitosan and β-glucans contribute to chemical functionality, toughness, and network cohesion. Minor constituents including proteins, glycoproteins, and lipids influence interfacial interactions, environmental stability, and biological functionality. Reactive hydroxyl, amino, and acetamide functional groups provide opportunities for hydrogen bonding, chemical modification, and composite integration [41].

In many fungal species, partial deacetylation of chitin generates chitosan, a cationic polysaccharide composed of glucosamine and N-acetylglucosamine units whose abundance depends on species, developmental stage, and environmental conditions [35,37,38]. Chitosan exhibits greater chemical reactivity and solubility than chitin because of the presence of free amino groups, allowing stronger interactions with water, ions, and other biopolymers [37,39,40]. The relative proportions of chitin, chitosan, glucans, and other cell-wall constituents therefore significantly influence the physicochemical behaviour of fungal-derived materials and may partly explain performance variations observed among MBCs produced from different fungal species [21,24,28]. The hierarchical organisation of fungal cell walls and the molecular components responsible for the structure and functionality of MBCs are illustrated in Figure 2.

To facilitate comparison of the major structural constituents of fungal cell walls and their contributions to material performance, the key components are summarised in Table 1.

As shown in Table 1, chitin primarily contributes structural rigidity and load-bearing capacity, whereas chitosan and β-glucans provide chemical functionality, network cohesion, and enhanced interfacial interactions. These polysaccharides collectively govern the structure–property relationships that underpin MBC performance.

Other than structural polysaccharides, fungal cell walls contain minor yet functionally important components such as proteins, glycoproteins, lipids, and melanin-associated molecules that contribute to cell-wall architecture, surface chemistry, environmental stability, and biological functionality. Cell-wall proteins and glycoproteins participate in crosslinking reactions and mediate interactions between polysaccharide networks, thereby influencing adhesion, elasticity, and matrix organisation [32,33,34]. Although present at lower concentrations, lipid components can alter hydrophobicity and moisture interactions, affecting the environmental stability of fungal biomaterials [21,22,24].

A key characteristic of fungal biopolymers is the abundance of reactive functional groups, including hydroxyl (–OH), amino (–NH_2_), and acetamide (–NHCOCH_3_) moieties, that facilitate intermolecular interactions, chemical modification, and interfacial bonding [36,37,39]. These functional groups promote extensive hydrogen-bond formation within and between polymer chains, contributing to the cohesive nature of mycelial networks and their ability to bind lignocellulosic substrates during composite fabrication [21,22,24]. Furthermore, the presence of chemically accessible hydroxyl and amino groups enables grafting, crosslinking, and surface functionalisation strategies that expand the range of potential engineering applications for mycelium-based materials [36,37,40].

### 2.2. Biosynthetic Pathways of Key Biopolymers

The remarkable functionality of fungal materials originates from tightly regulated biosynthetic pathways responsible for the assembly, organisation, and remodelling of chitin, chitosan, and glucan networks within the growing hyphal wall [33,34,42]. The synthesis and remodelling of these biopolymers occur continuously during hyphal extension, enabling fungi to simultaneously achieve structural integrity and adaptability under changing environmental conditions [32,33,34].

Chitin biosynthesis is mediated by membrane-associated chitin synthase enzymes that catalyse the polymerisation of uridine diphosphate N-acetylglucosamine (UDP-N-acetylglucosamine) into linear β-(1 → 4)-linked polysaccharide chains, which subsequently self-assemble into microfibrillar structures [34,42,43]. Multiple chitin synthase isoenzymes are typically present within fungal genomes, enabling precise regulation of polymer deposition during cell-wall expansion, branching, septum formation, and stress adaptation [33,43,44]. The organisation and spatial distribution of newly synthesised chitin strongly influence fibrillar architecture and contribute directly to mechanical reinforcement within fungal materials [22,33,34].

Chitosan is generated primarily through enzymatic deacetylation of chitin by chitin-deacetylases, which remove acetyl groups from N-acetylglucosamine residues and create glucosamine-rich polymer segments [37,38,45]. The extent of deacetylation depends on fungal species, developmental stage, environmental conditions, and enzymatic activity, resulting in considerable variability in polymer chemistry and functionality [38,40,45]. As amino groups generated during deacetylation increase polymer reactivity and intermolecular interactions, chitosan content is a critical determinant of fungal material properties including hydrophilicity, charge density, and chemical modifiability [37,39,40].

β-Glucans are synthesised by glucan synthase complexes located in the plasma membrane, producing predominantly β-(1 → 3)-linked backbones with β-(1 → 6)-linked branching structures that form the principal matrix surrounding chitin microfibrils. The degree of branching and molecular organisation of glucans strongly influences cell-wall porosity, flexibility, and network connectivity [32,33,34]. Through extensive physical interactions with chitin and other wall constituents, glucans contribute to the formation of hierarchical composite architectures that ultimately govern material performance, particularly toughness, flexibility, and moisture-transport behaviour [21,22,24].

Several molecular characteristics of fungal polysaccharides are particularly important for determining material behaviour, including degree of acetylation (DA), molecular weight, branching density, and crystallinity [36,37,40]. The degree of acetylation regulates charge distribution, intermolecular hydrogen bonding, solubility, and moisture affinity, thereby influencing both processing characteristics and final material properties [37,39,46]. Similarly, molecular weight and crystallinity affect chain mobility, stiffness, tensile behaviour, thermal resistance, and degradation kinetics, making these parameters key structure-property descriptors in fungal biomaterials [22,35,36].

### 2.3. Contribution of Fungal Polymers to Material Performance

The mechanical behaviour of mycelium-based materials arises from the synergistic interactions between fungal cell-wall polymers, hyphal network architecture, substrate constituents, and processing-induced microstructural features. At the molecular level, chitin microfibrils, glucan matrices, and associated cell-wall components contribute to the structural framework of fungal biomass; however, the macroscopic properties of MBCs emerge from the integrated composite system rather than from any individual polymer component alone [21,22,24]. Chitin functions as the principal reinforcing phase owing to its semi-crystalline structure and high intrinsic stiffness, while glucans contribute matrix-like behaviour that distributes stress and enhances structural cohesion [22,24,32,33,34]. These characteristics are generally believed to contribute to the mechanical integrity of fungal networks; however, the strength and stiffness of complete MBCs are also strongly influenced by porosity, density, substrate composition, fungal species, and processing conditions. The combination of rigid chitin fibrils and more compliant glucan networks is thought to contribute to the balance between strength, flexibility, and toughness observed in fungal tissues. In complete composites, however, these properties additionally depend on the organisation of the hyphal network and its interactions with surrounding substrate particles [21,22,24].

The density, orientation, and interconnectivity of hyphal networks strongly influence tensile, compressive, and flexural performance because stress transfer occurs through continuous fungal polymer networks extending throughout the material volume [21,24,30]. Enhanced glucan–chitin interactions increase network integrity and resistance to deformation, thereby improving load-bearing capacity and crack-arresting mechanisms [22,24,33]. Consequently, fungal polymer composition is increasingly recognised as an important contributor to MBC performance. However, mechanical reliability ultimately reflects the combined effects of polymer chemistry, fungal growth behaviour, interfacial bonding, composite microstructure, and post-processing treatments [21,27,28].

The abundance of hydroxyl and amino functional groups within fungal polysaccharides contributes to moisture sorption behaviour at the molecular level. Nevertheless, the moisture sensitivity of complete MBCs is additionally governed by factors such as porosity, density, surface characteristics, and the distribution of fungal biomass throughout the composite [36,37,39]. Moisture uptake can affect dimensional stability, mechanical performance, thermal conductivity, and long-term durability, making water–polymer interactions a key consideration in mycelium material design [24,27,30]. Differences in polymer composition, degree of acetylation, fungal species, and cell-wall organisation therefore contribute significantly to the moisture sensitivity observed among different mycelium-based composites [24,28,30].

Fungal cell-wall polymers contribute to thermal stability through their molecular structure and intermolecular interactions. However, the thermal response of complete MBCs depends not only on fungal biomass composition but also on substrate chemistry, composite architecture, density, and processing history [22,35,36]. Chitin-rich domains generally exhibit enhanced resistance to thermal degradation compared with amorphous polymer regions because of their higher crystallinity and stronger intermolecular interactions [35,36,37]. As a result, the chemical composition and molecular architecture of fungal polymers play a central role in determining the thermal response, serviceability, moisture resistance, and overall functional performance of MBCs across a wide range of applications [22,24,27]. Accordingly, the relationships between fungal polymer chemistry and MBC performance should be interpreted as contributory rather than strictly deterministic, with macroscopic properties emerging from multiscale interactions across the composite system.

## 3. Chemical Transformation of Complex Polymer Substrates

### 3.1. Composition of Polymeric Feedstocks

The production of MBCs relies predominantly on lignocellulosic feedstocks, which represent the most abundant renewable polymer resources on Earth and provide both structural support and carbon sources for fungal growth [47,48,49]. These substrates are typically derived from agricultural residues, forestry by-products, and industrial biomass wastes and consist primarily of cellulose, hemicellulose, and lignin arranged within complex hierarchical architectures that strongly influence fungal colonisation and substrate transformation [50,51].

Cellulose is the dominant structural polysaccharide in most plant-derived feedstocks and consists of linear β-(1 → 4)-linked D-glucose chains assembled into highly ordered microfibrils containing both crystalline and amorphous domains. Extensive intra- and intermolecular hydrogen bonding between cellulose chains generates semi-crystalline structures with high tensile strength, mechanical stiffness, and chemical stability, making cellulose a key reinforcing component within natural and engineered composite systems [47,52,53]. The degree of crystallinity, fibril organisation, and accessibility of cellulose significantly influences fungal colonisation efficiency and enzymatic degradation kinetics during mycelium composite fabrication [48,49,51].

Hemicellulose constitutes a heterogeneous group of amorphous branched polysaccharides that surround cellulose microfibrils and contribute to the structural organisation of plant cell walls [54,55,56]. Unlike cellulose, hemicellulose contains multiple sugar monomers including xylose, mannose, arabinose, galactose, and glucose arranged in highly branched architectures that are generally more susceptible to enzymatic degradation [49,54,56]. Consequently, hemicellulose often serves as one of the first substrate fractions accessed and metabolised during fungal colonisation, influencing nutrient availability and substrate remodelling dynamics [49,51].

Lignin is a highly complex aromatic polymer composed primarily of p-coumaryl, coniferyl, and sinapyl alcohol-derived phenylpropanoid units that form extensive crosslinked networks throughout plant tissues. The recalcitrant nature of lignin arises from its heterogeneous structure and abundance of carbon–carbon and ether linkages, which contribute significantly to resistance against biological and chemical degradation [57,58,59]. As a result, lignin acts as both a physical barrier and a complex carbon reservoir, making lignin-rich substrates particularly influential in determining fungal colonisation behaviour and degradation pathways [49,60,61].

Beyond conventional lignocellulosic biomass, fungi can colonise a wide range of agricultural and industrial feedstocks including straw, hemp hurds, corn stover, sawdust, cotton residues, paper waste, textile fibres, food processing residues, and other polymer-rich by-products that provide diverse chemical compositions and structural characteristics [21,24,28]. The physicochemical properties of these substrates, including particle size, chemical composition, moisture content, porosity, and surface chemistry, strongly influence fungal growth behaviour and the resulting material performance of mycelium-based composites [24,28,31].

### 3.2. Enzymatic Degradation Mechanisms

The ability of filamentous fungi to transform complex polymeric substrates is largely attributed to their extensive extracellular enzymatic machinery, which enables the deconstruction and utilisation of lignocellulosic biomass under environmentally benign conditions [48,49,60]. These enzymatic processes involve coordinated hydrolytic and oxidative reactions that progressively convert insoluble plant polymers into assimilable molecular intermediates while simultaneously altering substrate structure and chemistry [49,61,62]. As multiple enzyme systems operate simultaneously during fungal colonisation, the principal enzymes involved in lignocellulosic substrate degradation are summarised in Table 2.

The enzymes listed in Table 2 act synergistically to modify cellulose, hemicellulose, and lignin fractions, thereby influencing nutrient availability, substrate porosity, and ultimately the physical characteristics of the resulting composite.

Cellulose degradation is primarily mediated by cellulase systems composed of endoglucanases, exoglucanases, and β-glucosidases, whereas hemicellulose degradation involves diverse enzyme families including xylanases, mannanases, arabinofuranosidases, and esterases. In contrast, lignin transformation depends predominantly on oxidative enzymes such as laccases and peroxidases, which generate reactive radicals capable of depolymerising aromatic structures. Together, these enzymatic systems enhance substrate accessibility, increase porosity, and facilitate fungal colonisation and biomass production [48,49,56,60,61,62,63,64,65,66].

Through combined hydrolytic and oxidative pathways, fungal enzymes progressively depolymerise complex plant polymers into chemically reactive intermediates including glucose, xylose, oligosaccharides, aromatic aldehydes, organic acids, and phenolic compounds [49,60,61]. These degradation products not only support fungal metabolism and biomass generation but also participate in secondary chemical transformations that influence interfacial bonding, fungal polymer synthesis, and composite development [21,24,25].

### 3.3. Interfacial Bonding and Composite Formation

The successful formation of MBCs depends on the establishment of strong interfacial interactions between fungal hyphae and substrate particles throughout the colonisation process [21,22,24]. Unlike conventional composites that often rely on synthetic binders, mycelium composites are formed through biologically driven self-assembly mechanisms that integrate fungal polymers directly with substrate constituents [21,24,26].

Hydrogen bonding is widely proposed as an important contributor to interfacial adhesion in MBCs because both fungal cell-wall polymers and plant-derived substrates contain abundant hydroxyl, amino, and acetamide functional groups capable of forming intermolecular interactions. However, the relative contribution of hydrogen bonding has not always been directly quantified in complete composite systems and is often inferred from the chemistry of the interacting biopolymers [22,37,39]. These non-covalent interactions facilitate intimate contact between fungal hyphae and substrate surfaces and contribute significantly to composite cohesion and mechanical integrity [21,22,24].

Electrostatic interactions have similarly been proposed as potential contributors to fungal–substrate adhesion through charged functional groups present in chitosan-rich cell-wall regions, proteins, and modified substrate components generated during fungal degradation processes. Nevertheless, direct experimental evidence quantifying the role of electrostatic interactions in complete MBCs remains limited [37,39,40]. Variations in pH, ionic strength, and degree of polymer deacetylation can influence these interactions and therefore affect the microstructural organisation of fungal–substrate interfaces [39,40,46].

In contrast, physical interactions involving hyphal penetration, wrapping, bridging, and entanglement around substrate particles have been directly observed in numerous microscopy-based studies and are widely recognised as key contributors to composite formation. These mechanisms create a mechanically interlocked network throughout the developing composite and represent one of the most experimentally supported modes of fungal–substrate integration [21,22,24]. Continuous hyphal growth bridges neighbouring particles and generates interconnected architectures that have been experimentally linked to enhanced cohesion and load transfer within MBCs [21,24,28].

As colonisation progresses, fungal biomass and residual substrate components become integrated into an interpenetrating network in which biological and plant-derived polymers coexist across multiple length scales [22,24,25]. This hierarchical organisation gives rise to a chemically and structurally integrated composite system whose properties emerge from synergistic interactions between fungal cell-wall polymers, transformed substrate constituents, and the resulting microstructural architecture [21,24,28]. Overall, current evidence suggests that fungal–substrate bonding arises from a combination of experimentally observed physical interlocking mechanisms and physicochemical interactions that are often inferred from polymer chemistry. Further studies employing spectroscopic, microscopic, and interfacial characterisation techniques are required to quantify the relative contributions of these mechanisms to composite performance.

### 3.4. Chemical Evolution During Fungal Colonisation

Fungal colonisation is accompanied by continuous chemical transformation of the substrate as extracellular enzymes selectively depolymerise cellulose, hemicellulose, and lignin while simultaneously generating new fungal biomass [49,60,61]. These processes result in substantial modifications to substrate composition, functional group distribution, and molecular architecture over the course of material formation [24,28,31].

Numerous spectroscopic, thermal, and compositional studies have demonstrated reductions in carbohydrate and lignin fractions together with changes in hydroxyl, carbonyl, and aromatic functional groups during fungal degradation and substrate utilisation [24,31,60]. Selective removal of hemicellulose and partial lignin modification often increases substrate porosity and surface accessibility, facilitating deeper fungal penetration and enhanced biological integration [49,51,61].

Simultaneously, fungal metabolism converts assimilated carbon sources into new structural biopolymers including chitin, chitosan, β-glucans, proteins, and extracellular matrix components that progressively accumulate within the material [24,32,33,34]. Consequently, the relative proportion of fungal-derived polymers increases throughout colonisation, gradually shifting the system from a substrate-dominated architecture toward a biologically integrated composite structure [21,22,25].

The final composition of MBCs therefore reflects a dynamic balance between substrate degradation, fungal polymer biosynthesis, and interfacial network formation rather than a simple mixture of biomass and fungal cells [24,25,28]. Understanding this chemical evolution is essential for establishing process–structure–property relationships and developing predictive strategies to optimise material performance through feedstock selection, fungal strain engineering, and process control [21,24,31].

## 4. Processing Strategies and Material Formation

### 4.1. Growth Conditions and Bioprocess Parameters

The formation and performance of MBCs are strongly governed by environmental and bioprocess parameters that regulate fungal metabolism, hyphal extension, substrate utilisation, and cell-wall biosynthesis throughout cultivation, thereby influencing the resulting microstructure and functional properties of the composite [21,24,28]. As mycelium materials are generated through biological growth rather than conventional manufacturing, processing conditions directly influence the chemical composition, microstructure, and functional properties of the final composite [22,26,27].

Temperature is among the most critical factors affecting fungal physiology because enzymatic activity, nutrient assimilation, respiration, and polymer biosynthesis are all highly temperature-dependent processes [24,28,63]. Most fungal species used in mycelium composite production exhibit optimal growth within moderate temperature ranges, typically between 20 and 30 °C, where metabolic activity and hyphal extension rates are maximised without causing thermal stress [21,24]. Deviations from optimal temperatures can alter cell-wall composition, growth kinetics, and substrate colonisation efficiency, ultimately affecting material homogeneity, microstructural development, and performance [27,28,63].

Humidity and water availability are equally important because fungal growth relies on sufficient moisture to support nutrient transport, enzymatic reactions, and cellular expansion [24,28]. Excessively low moisture levels can restrict colonisation and reduce biomass production, whereas excessive moisture may impair oxygen transport and increase the risk of contamination or undesirable microbial competition [21,24,27]. Consequently, moisture control plays a central role in determining the density, morphology, and consistency of fungal networks throughout the substrate matrix [24,28,31].

The pH of the cultivation environment significantly influences fungal metabolism, enzyme secretion, nutrient solubility, and polymer biosynthesis by affecting biochemical reaction equilibria and enzyme functionality [33,49,63]. Variations in pH can alter the production of lignocellulose-degrading enzymes and influence the chemical interactions that occur at fungal–substrate interfaces during composite formation [31,49,61].

Oxygen availability is another critical processing variable because filamentous fungi depend on aerobic respiration to generate the energy required for growth, biomass production, and extracellular enzyme synthesis [21,24,63]. Restricted oxygen transport within densely packed substrates can reduce metabolic activity and lead to non-uniform colonisation, whereas adequate aeration promotes extensive hyphal development, improved substrate colonisation, and more homogeneous material formation [21,27,28].

Nutrient composition, including carbon-to-nitrogen ratio, mineral content, and the availability of assimilable carbon sources, directly affects fungal growth kinetics and cell-wall polymer biosynthesis [24,28,33]. Nutrient-rich substrates generally promote rapid biomass accumulation and increased production of structural polymers such as chitin and β-glucans, while nutrient limitations may alter hyphal morphology and trigger adaptive physiological responses [21,32,33,34]. Consequently, nutrient availability influences both growth rate and the deposition of fungal polymers that ultimately determine the microstructure and performance of mycelium-based composites [22,24,28].

### 4.2. Growth-Induced Material Fabrication

A unique characteristic of mycelium-based materials is that fabrication occurs simultaneously with biological growth, enabling material formation through self-organised biosynthesis rather than conventional assembly processes [21,22,26]. During cultivation, fungal hyphae continuously expand, branch, and interconnect throughout the substrate, progressively generating a cohesive composite architecture through biological binding mechanisms [22,24,25].

One of the most widely adopted fabrication approaches is in situ mould-based growth, in which inoculated substrates are placed within predefined moulds and allowed to colonise until sufficient structural integrity is achieved [21,24,28]. This strategy enables complex geometries to be produced directly during growth without requiring extensive machining, minimising material waste, or necessitating secondary shaping operations [21,26,27]. As the mycelium colonises the mould cavity, fungal biomass integrates substrate particles into a continuous network that conforms precisely to the mould geometry [22,24,28].

The fabrication process is fundamentally driven by the self-assembly behaviour of fungal hyphae, which spontaneously form interconnected three-dimensional networks through branching, anastomosis, and directional growth [22,24,33]. These self-organising processes generate hierarchical architectures spanning multiple length scales, from nanoscale cell-wall structures to macroscale material geometries [21,22,24]. The resulting three-dimensional network functions both as a biological scaffold and as a natural reinforcing phase, facilitating stress transfer and structural integration throughout the composite, producing structurally integrated materials without the need for synthetic adhesives or binders [24,25,26].

As growth and fabrication occur simultaneously, material architecture can be tailored through control of fungal species, substrate composition, growth conditions, and mould design, providing an unusually versatile platform for sustainable material manufacturing [21,27,28].

### 4.3. Post-Processing and Densification

Following biological growth, mycelium-based materials commonly undergo post-processing treatments intended to stabilise the structure, terminate biological activity, and enhance engineering performance [21,24,28]. These treatments can significantly alter the microstructure, polymer organisation, and interfacial interactions within the composite, thereby influencing final material properties [22,24,27].

Thermal drying is typically employed as the first post-processing step to remove residual moisture and halt fungal metabolism while preserving the overall composite architecture [21,24]. Moisture removal promotes additional intermolecular interactions between fungal and substrate polymers through closer molecular packing and enhanced intermolecular hydrogen bonding [22,24,39]. Furthermore, drying improves dimensional stability and reduces the susceptibility of materials to microbial degradation during storage and service [21,27,28].

Hot pressing is frequently applied to increase material density and improve mechanical performance through the combined effects of heat and pressure [21,24,64]. Elevated temperatures soften certain substrate constituents and facilitate polymer rearrangement, whereas applied pressure compresses void spaces and promotes intimate contact between fungal biomass and substrate particles [22,24,64]. These changes often result in enhanced stiffness, compressive strength, and dimensional stability compared with untreated composites. Representative studies have demonstrated that hot-pressing and densification treatments can increase compressive strength from values below 0.1 MPa in highly porous systems to greater than 1–4 MPa in densified materials, depending on fungal species, substrate selection, and processing conditions [21,24,27].

Mechanical compression without elevated temperatures can likewise increase composite density through pore collapse and structural consolidation [24,28,64]. Compression improves stress transfer efficiency by reducing interparticle distances and increasing the number of contact points throughout the material network [21,22,24]. Consequently, densification treatments commonly lead to reduced porosity, increased hydrogen-bonding density, and stronger fungal-substrate interfacial interactions [22,24,39].

At the molecular level, these post-processing methods promote greater polymer packing efficiency and facilitate stronger intermolecular associations among chitin, glucans, cellulose, and lignin-derived constituents [21,22,24]. The resulting structural consolidation contributes significantly to improvements in mechanical properties, dimensional stability, and long-term performance [24,27,28].

### 4.4. Processing–Structure Relationships

**Figure 3:**
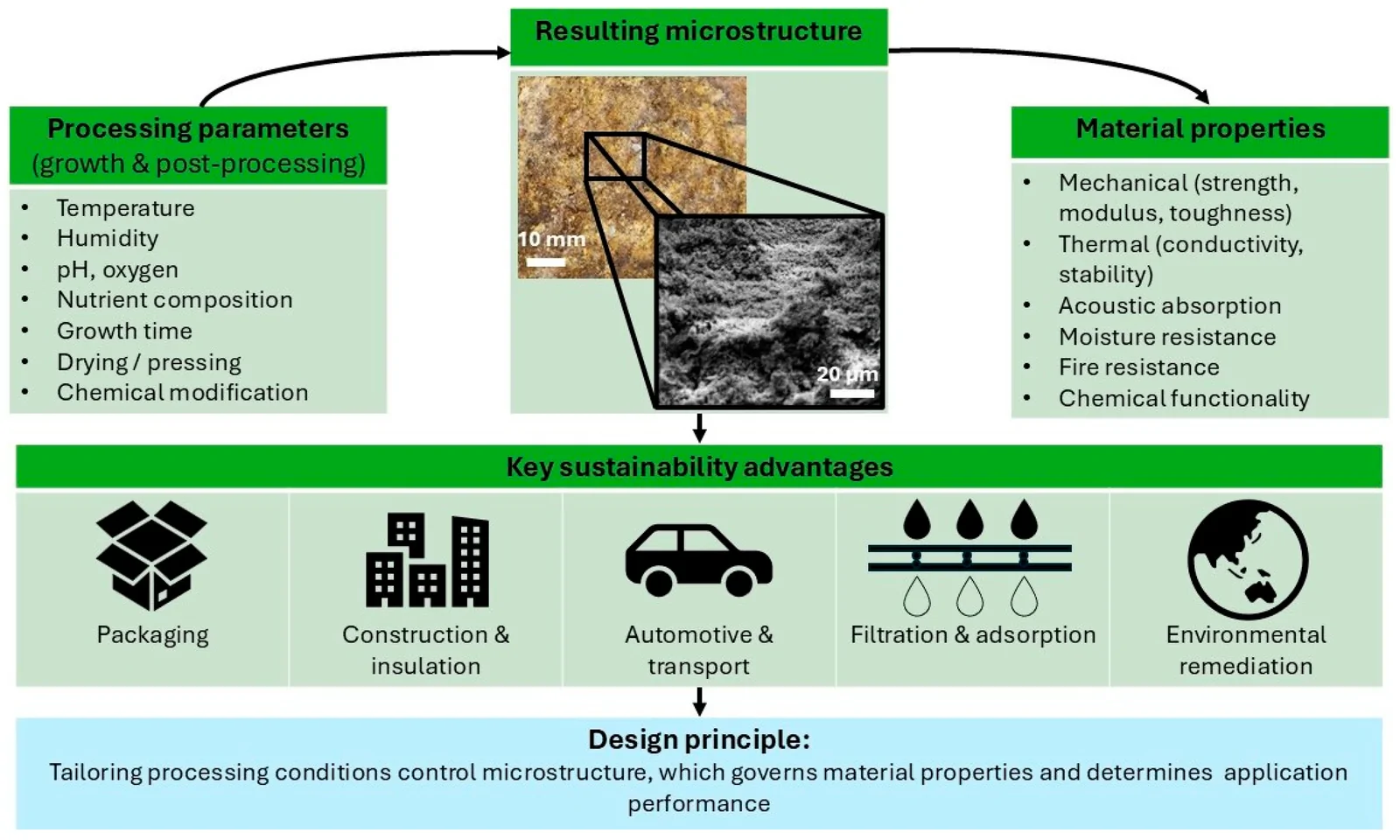
Processing–structure–property relationships in mycelium-based composites. Cultivation and post-processing parameters influence the development of microstructure, including hyphal density, pore architecture, substrate integration, and network connectivity. These structural features control mechanical, thermal, acoustic, moisture-related, fire-resistant, and functional properties that ultimately determine application performance.

The final properties of mycelium-based composites are strongly governed by processing-induced changes in microstructure, polymer organisation, and interfacial architecture, making processing–structure relationships central to material design [21,22,24,28,30]. The influence of cultivation and post-processing conditions on composite development is multifactorial. The processing–structure–property framework governing MBC performance is illustrated in Figure 3, while the principal relationships are summarised in Table 3 [21,22,24,28,30].

As summarised in Table 3, optimisation of MBC performance often involves balancing competing effects. For example, densification treatments typically increase bulk density while reducing porosity, resulting in substantial improvements in compressive strength and stiffness. However, corresponding increases in thermal conductivity are frequently observed because of the reduced volume of insulating air-filled voids within the material structure [24,27,30,64].

Post-processing operations modify composite microstructure by reducing porosity, increasing network density, and strengthening intermolecular interactions. Consequently, densification treatments generally improve compressive strength, stiffness, and dimensional stability while reducing structural heterogeneity [21,22,24,27,30,64].

Processing-induced modifications also influence thermal performance because porosity, density, and polymer organisation strongly affect heat transfer mechanisms within the material [22,27,30]. Highly porous composites generally exhibit lower thermal conductivity and superior insulation performance, whereas densified structures often display improved mechanical properties but increased thermal transport due to reduced air-filled void content [21,24,30]. Therefore, material optimisation frequently involves balancing competing requirements between density, mechanical performance, thermal insulation, and durability through careful control of cultivation and post-processing parameters [27,28,31]. Collectively, these processing-induced modifications establish the process–structure–property relationships that govern the performance of MBCs and provide a foundation for subsequent material optimisation strategies discussed in Section 5.

## 5. Chemical Modification and Material Optimisation

The chemical versatility of fungal biomaterials arises from the abundance of hydroxyl, amino, and acetamide functional groups present within chitin-, chitosan-, and glucan-rich cell walls. These reactive functional groups can be exploited for material modification. Numerous modification strategies have therefore been explored to tailor mechanical performance, wettability, thermal stability, adsorption behaviour, and compatibility with other material systems. The major approaches reported in the literature are summarised in Table 4.

**Figure 4:**
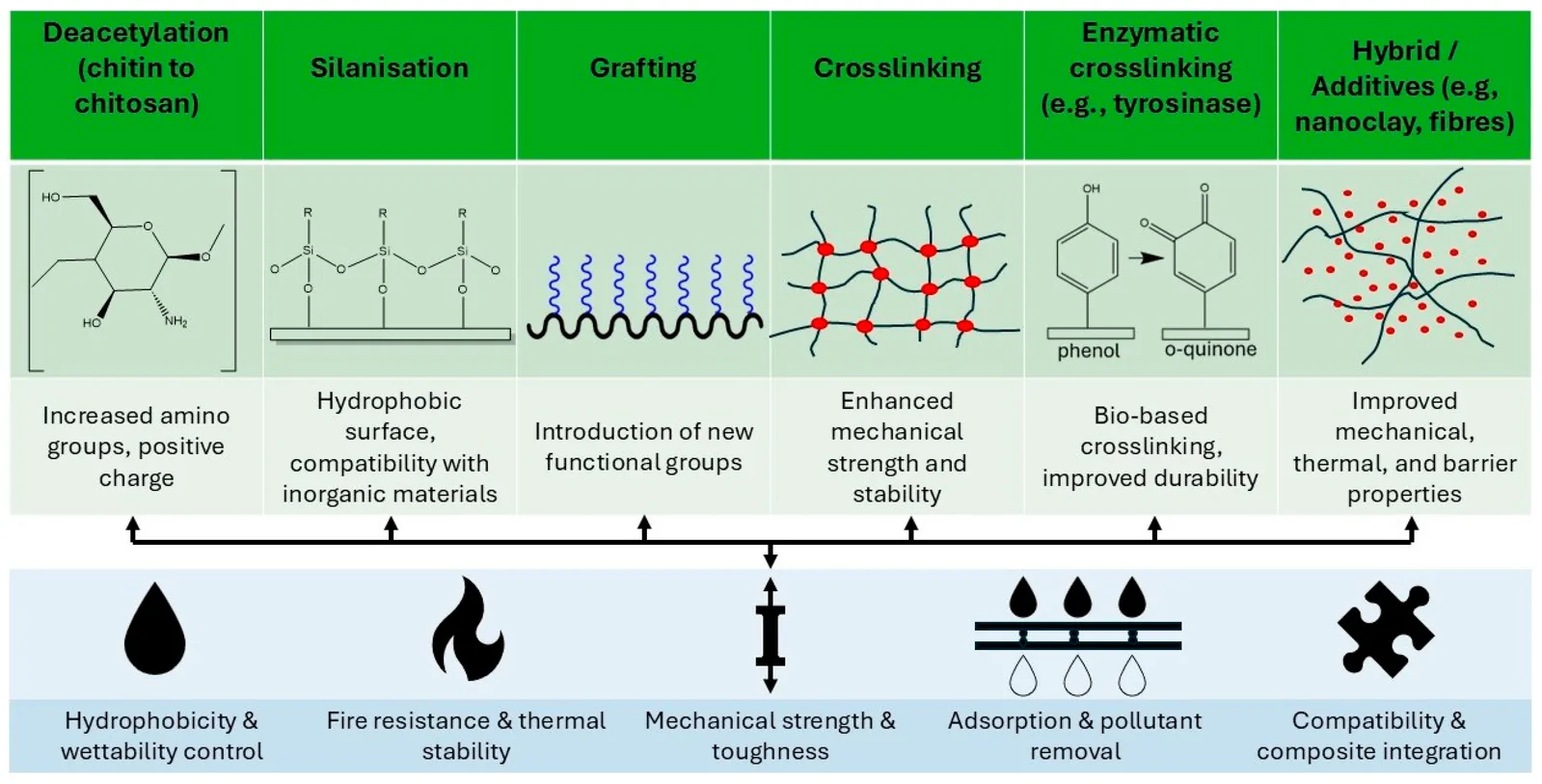
Major chemical modification pathways and functional outcomes in mycelium-based materials. Deacetylation, silanisation, grafting, crosslinking, enzymatic crosslinking, and hybrid additive incorporation modify the chemical structure and surface functionality of fungal biomaterials. These approaches can improve wettability control, thermal stability, fire resistance, adsorption capacity, mechanical performance, and compatibility with composite systems.

While deacetylation and grafting primarily alter surface chemistry, crosslinking and hybrid reinforcement approaches are generally aimed at improving mechanical durability and multifunctionality. Major chemical modification routes used to tailor the performance of mycelium-based materials and their corresponding functional outcomes are summarised in Figure 4.

Modification of surface chemistry can significantly influence wettability, mechanical performance, adsorption behaviour, and interfacial bonding. By altering this surface chemistry, it has been shown to improve the mechanical properties of mycelium by removing weaker parts of the chain while improving interchain hydrogen bonding and surface polarity [65].

### 5.1. Chemical Functionalisation of Mycelium Materials

One of the most widely investigated chemical modifications of fungal biomaterials is the deacetylation of chitin to produce chitosan [66]. Deacetylation removes acetyl groups (-COCH) from chitin, generating additional amino functionalities and increasing polymer reactivity. Typically, chitin is deacetylated using alkaline solutions, particularly NaOH, which remove acetyl groups and produce sodium acetate as a by-product. The resulting changes in intermolecular interactions can enhance chemical reactivity, adsorption performance, and thermal stability [67,68]. As chitosan possesses additional amino functionalities relative to chitin, it provides additional opportunities for chemical modification. It should be noted, however, that many reported functionalisation strategies have been demonstrated primarily in isolated chitin or chitosan systems rather than in complete MBCs [67,68]. Consequently, direct translation of these results to whole-mycelium materials should be interpreted with appropriate caution.

Grafting of mycelium allows for modification of the polymeric chain similar to other methodologies in which the surface functional groups are exploited as reaction sites [70,71]. By grafting a branch onto the surface, a broad range of different properties can be obtained, allowing new properties to be achieved. A representative example of direct whole-mycelium modification is the development of a microplastic adsorbent through treatment of the mycelium network with 2,3-epoxypropyl-trimethylammonium chloride (EPTAC) to alter the charge of the mycelium network’s surface [72]. The modified fibrous network demonstrated efficient microplastic adsorption from solution but was found to be limited without surface modification compared to the samples grafted with EPTAC which had approximately twice the adsorption capacity. This performance improvement was attributed to the combination of change in the surface charge, and changes to morphology trapping the microplastic particles. Similarly, Song et al. [70] demonstrated that the adsorption capacity of *Ganoderma lucidum* mycelium is strongly influenced by its morphology, with linear mycelium exhibiting the highest adsorption performance due to its larger specific surface area, greater pore volume, and more extensive fibrous network structure. The authors further showed that microplastic capture occurs through a combination of physical interception, electrostatic interactions, and chemical interactions involving amino, carboxyl, and hydroxyl functional groups on the mycelial surface, highlighting the importance of both surface chemistry and morphology in adsorption performance. Despite the promising performance improvements reported for deacetylation and grafting strategies, several limitations remain. Deacetylation typically requires concentrated alkaline solutions and generates chemical waste streams that must be appropriately managed, potentially reducing the environmental advantages of bio-based materials. Similarly, grafting approaches can substantially improve adsorption performance and interfacial functionality; however, increased processing complexity, chemical consumption, and treatment costs may limit large-scale implementation. Consequently, the selection of functionalisation strategies should balance performance benefits against economic, environmental, and manufacturing considerations.

Overall, chemical modification strategies reported in the literature span two distinct categories: modifications performed directly on whole-mycelium materials or completed MBCs, and modifications demonstrated primarily on isolated fungal biopolymers such as chitin and chitosan. While the latter provide valuable insight into potential functionalisation routes, further research is required to establish how these modifications influence the behaviour of complete composite systems.

### 5.2. Crosslinking Strategies

Silane coupling agents are commonly used for the surface functionalisation of organic materials by forming self-assembled monolayers, allowing for compatibility with inorganic materials depending on the functional groups present on the silane. Mycelium has been successfully employed as a biological template for the synthesis of silicon oxide nanofibers via bonding aminopropyl triethoxysilane (APTES) onto the mycelium hyphae and removal of the organic components via high-temperature treatment [73]. The APTES-modified mycelium structures were also shown to have high thermal resistance suggesting potential applications in insulation and fireproofing.

Mycelium-based aerogels containing different silanes were developed to separate oil from water [74]. In this work, the addition of silane coupling agents was shown to improve hydrophobicity through replacement of hydrophilic hydroxyl groups with hydrophobic surface functionalities. The aerogels were shown to exhibit good mechanical resilience enduring 40% compressive strains explained by the anisotropic structure. The removal of oil from a solution containing water was shown to separate the oil from water selectively, with 99% efficiency, leaving behind clean water.

Elastomer-like gamma radiation-mutated mycelium mats were produced using a series of different modifications (acetylation, benzoylation and silanisation) [75]. Mycelium was treated via gamma radiation to improve the growth rate and mycelial mat-formatting capabilities. The grown mycelium mats underwent a treatment with a NaOH solution to help the different chemical treatments penetrate the network better before having the surface modification performed. After surface modification, the mats were submersed in a coating solution containing poly(ethylene glycol) (PEG), corn zein and ethanol. From this surface modification treatment, the developed mats were shown to have an improvement in the elongation rate with the modified samples doubling the elongation of the base material.

Enzymatic crosslinking systems are a biobased approach as an alternative to more traditional synthetic crosslinking systems. Enzymes which target polyphenols such as tyrosinase work by converting phenol groups into the more reactive o-quinone which works as the cross linker which can preferentially react with the amine groups present in chitosan [76]. Among the available modification approaches, silanisation and crosslinking generally provide substantial improvements in moisture resistance, thermal stability, and compatibility with hybrid systems [25]. However, these benefits are often accompanied by higher material costs and additional processing requirements. Furthermore, some crosslinking strategies may reduce biodegradability or recyclability by creating more chemically stable networks. Consequently, optimisation requires balancing long-term durability with the sustainability advantages that motivate the development of MBCs [25].

### 5.3. Additives and Hybrid Systems

A major motivation for incorporating mycelium into composite materials is its ability to form interconnected fibrous networks that combine low density with effective particle binding and stress-transfer capability. These characteristics arise from the complex hyphal architecture, which provides physical entanglement and multiple bonding interactions throughout the composite network [77]. Although these characteristics are advantageous, mycelium-based materials can benefit from having these properties enhanced.

Instead of having mycelium as the substrate or filler of a composite material, it is possible to have mycelium as the primary matrix with additional content to improve the bulks’ performance. Mycelium- and lignocellulose-based composite materials have been garnering strong interest due to the potential to replace non-bio-based materials, particularly in applications such as foams, and insulation panels [21]. Mycelium bonds to the lignocellulose content via a combination of physical, chemical and enzymatic bonding [78]. Alternatively, inorganic materials such as clay minerals represent a promising alternative to conventional lignocellulosic reinforcement for the development of mycelium-based composites. The incorporation of nanostructured clays can generate bio-ceramic hybrid materials in which the inorganic phase modifies hyphal growth and network architecture while enhancing durability and environmental resistance. Halloysite nanotubes have been demonstrated to functionalise fungal mycelia, improving hyphal spreading and regulating sorption behaviour, highlighting the versatility of clay-based additives in mycelium engineering [79]. Nevertheless, while nanoclay incorporation may improve durability and functionality, excessive additions can negatively affect mycelial colonisation and reduce the mechanical properties of the resulting composite through disruption of the fungal fibre network [80].

The addition of inorganic nanoparticles into mycelium allows for functional properties that would not be possible with purely natural materials. Gold nanoparticles (AuNP) were successfully grafted onto mycelium for use as a biofilters for mercury [81]. The impact on the cell-wall biochemistry after the deposition of coated AuNPs onto the mycelium surface was shown to vary quite considerably depending on time, concentration and coating used with all of them causing stress to the growth but were still viably deposited [81,82].

Other metallic nanoparticles have been incorporated into mycelium for similar uses. Silver nanoparticle-containing mycelium composites have shown promise in catalytic degradation of dyes, particularly methylene blue, along with having potential use as antibacterial products [83]. The influence of substrate selection on the mechanical performance of MBCs is illustrated through representative examples summarised in Table 5.

As shown in Table 5, a broad range of materials have been explored as the substrate for MBCs with a particular focus towards biological and agricultural waste [84,87,88,89]. MBCs were produced with natural reinforcing agents acting as a filler to improve the materials’ compression strength [90]. The mechanical performance has been shown to vary significantly between different MBCs which is due to the complex nature of the developed materials and also differences in manufacturing and modifications. The biocomposites were produced by incubating the mycelium with different substrates containing cotton stalk, wheat bran and carbonate sand in different loadings. From the work, it was shown that the biocomposites produced created a complex network where the hyphae of the mycelium would grow and intertwine between the wheat bran and cotton stalks, leading to a denser network with the compressive strength improving with increasing concentration of filler. While the substrate for growth is a major factor for consideration, it is not the only parameter which needs to be considered when tailoring the properties of MBC materials, how the mycelium is processed (pressed vs. unpressed) and skin thickness also have an impact [24,91].

Three-dimensional-printable mycelium-based composite materials containing wood flour and nanoclay were developed [92]. In this work, a hydrogel containing bamboo wood flour and a hydrogel containing bentonite were printed into complex 3D structures which were used as a support on the surface of which to grow mycelium. The final composite materials containing mycelium were shown to have improved performances in comparison to the material without. With improved hydrophobicity being attributed to the mycelium surface (76.9 ± 4.7° to 132.4 ± 11.2°), improvements were achieved in the thermal insulation properties and mechanical properties, with an over 700% increase in the Young’s modulus and 140% increase in the ultimate tensile strength. Hybrid reinforcement strategies currently appear among the most promising routes for improving the engineering performance of MBCs because they simultaneously address mechanical limitations and functional requirements [93,94]. Nevertheless, increasing additive content may negatively affect fungal colonisation, increase composite density, reduce biodegradability, and introduce additional supply-chain and processing complexity [93,94]. Therefore, future development should focus on identifying reinforcement levels that maximize performance gains without compromising biological growth or sustainability objectives.

### 5.4. Tailoring Functional Properties

Mycelium is typically hydrophobic owing to the presence of hydrophobins within fungal cell walls [95,96]. This property is advantageous for applications requiring moisture resistance and environmental durability; by incorporating mycelium onto the surface of biobased materials, it is shown to hinder the impact of water exposure; however, this hydrophobicity is diminished by continuous exposure [97] leading to saturation of the mycelium.

The wettability of mycelium has been shown to be tuneable via deacetylation of the surface and pulping with a NaOH/H_2_O_2_ solution [65]. From this treatment, the pulped mycelium materials were shown to have superior mechanical performance and improvement in swelling capacity compared to both the original mycelium and the deacetylated mycelium. These improvements were attributed to the pulping process by removing the amorphous regions, in turn improving the crystallinity and transforming the material from a porous network into a compact ordered sheet.

The wettability of mycelium foams has also been shown to be tuneable via treatment with water and room-temperature drying. The surface wettability was shown to be reversible via drying at elevated temperatures [97]. After pressing the mycelium foams into films, the samples were saturated with water before being allowed to dry in air at room temperature. From this wetting process, the water contact angle was shown to decrease significantly compared to the untreated samples which were proposed to be due to changes in the surface structure with the hyphae swelling leading to a denser surface.

Mycelium-based materials possess considerable potential as fire-resistant materials due to a combination of properties, particularly the charring process which mycelium undergoes when exposed to high temperatures [98,99]. The improvement in thermal stability has been proposed to be due to the deacetylation process as the thermal stability increased with increased conversion of chitin into chitosan and also hydroxyl-terminated polysaccharide segments [67]. Incorporation of this deacetylated mycelium into an epoxy network along with GFRP which was shown to delay the onset of ignition, reduce flaming intensity and improve the fire safety ranking due to the charring at the heat-exposed surface.

Composite boards have been produced by hot-pressing mycelium grown on mixtures of straw and food-processing residues [100]. By pressing the composite material, the mycelium acted as an adhesive, creating an alternative to commercial boards while also exhibiting excellent thermal properties, particularly fire resistance. From the testing, the boards were shown to be on par with other common boards used in construction showing promise in the future as a green alternative to current solutions; however, there are currently limitations due to poor water resistance [100].

Due to their favourable physicochemical properties, particularly their thermal and acoustic insulation performance, mycelium-based materials have been extensively investigated for construction applications [101], with a particular focus on concrete and bricks [102,103]. Mycelium as a filler for concrete is highly beneficial, particularly due to the production of calcium carbonate acting as a self-healing material [104].

Bricks containing mycelium were infused with a range of geopolymers to explore their viability in non-load-bearing walls [105]. A range of approaches were explored including using mycelium in the mortar, mycelium epoxy and geopolymer composites and partial replacement of fly ash with mycelium. The developed mycelium bricks had their properties tested including mechanical, fire resistance, water resistance and acoustic insulation. From the testing, it was found that NaOH-treated mycelium and ash-based geopolymer concrete performed as well as normal geopolymer concretes, showing their viability.

Tiles were developed by growing two different mycelium species onto waste bamboo fibres [106]. The produced tiles were tested for their thermal conductivity properties both indoors and outdoors before having their durability tested by exposing them to the environment for two months uncoated. From the testing, it was shown that the two different species had similar thermal performances; however, there was a significant difference in the durability testing with the sample containing *Ganoderma lucidum* outperforming the ones containing *Pleurotus ostreatus*, which was attributed to higher concentration of hydrophobins allowing the tiles to remain hydrophobic even when exposed to tropical conditions. After the durability testing was completed, the produced tiles were also shown to be biodegradable. Collectively, the reported studies demonstrate that chemical modification can substantially improve moisture resistance, fire performance, adsorption behaviour, and mechanical properties. However, no single modification strategy simultaneously maximises all performance metrics. Treatments that improve durability or fire resistance may increase processing complexity or reduce biodegradability, whereas highly sustainable approaches may provide more modest property enhancements. Consequently, future optimisation efforts will likely require integrated approaches combining chemical modification, targeted reinforcement, and process control to achieve application-specific performance requirements.

## 6. Applications of MBCs

### 6.1. Packaging Materials

Packaging is one of the most commercially mature applications of MBCs because fungal growth can consolidate lignocellulosic wastes into lightweight structures with mechanical and energy-absorption properties suitable for protective packaging applications [21,24,25,26,27]. The ability of mycelium to act as a natural adhesive through hyphal growth and interfacial bonding enables the production of moulded packaging components without the need for synthetic binders, thereby reducing dependence on petroleum-derived polymer foams [22,24,25,26,98]. The resulting cellular architecture contains interconnected pores that deform under load, allowing impact energy to be dissipated through pore collapse, network compression, and stress redistribution throughout the hyphal matrix [21,22,24,27].

An additional advantage of mycelium-based packaging is its favourable end-of-life profile. Unlike conventional expanded polystyrene and polyurethane foams, mycelium composites are largely composed of fungal biomass and renewable plant-derived polymers that can be biodegraded under composting or natural environmental conditions [1,21,25,26,27]. Furthermore, the use of agricultural and industrial waste streams as feedstocks aligns with circular economy principles by transforming low-value biomass into functional materials while reducing landfill disposal and resource consumption [25,26,27,28,98]. The combination of biodegradability, low density, moldability, and renewable feedstocks therefore positions MBCs as promising alternatives to conventional packaging materials.

### 6.2. Construction and Thermal Insulation

Building and insulation materials represent one of the most actively investigated applications of mycelium-based composites due to increasing demand for low-carbon construction materials and sustainable alternatives to conventional insulation products [21,24,26,27,28]. The thermal insulation performance of mycelium composites originates primarily from their highly porous structure, which traps air within interconnected voids and restricts heat transfer through the material [21,22,24,30,98]. As air possesses a significantly lower thermal conductivity than the surrounding biopolymer network, controlling pore size distribution and porosity becomes a key strategy for optimising insulation performance [21,22,24,27,30].

Processing conditions have a major influence on thermal behaviour because densification treatments such as hot pressing reduce pore volume while increasing the continuity of the solid phase, thereby affecting heat transport mechanisms. Highly porous materials generally provide superior thermal insulation performance, whereas denser materials typically exhibit improved mechanical performance but somewhat higher thermal conductivity [24,27,30,31,64].

Recent studies have additionally demonstrated that fungal bioprocessing can convert heterogeneous waste streams into insulation materials with excellent thermal stability and fire resistance. In particular, biomineralisation phenomena and char-forming behaviour associated with fungal polymers have been shown to improve high-temperature performance and contribute to fire-resistant insulation systems [21,22,24,27,98]. These characteristics make mycelium composites attractive candidates for non-load-bearing building components, interior architectural elements, insulation boards, architectural panels, and acoustic barriers.

### 6.3. Non-Structural and Automotive Applications

The low density and favourable specific mechanical properties of mycelium-based materials have generated interest in non-structural engineering applications including furniture, interior architectural products, partition systems, consumer goods, and automotive interior components. Through mould-based growth and targeted post-processing strategies, mycelium composites can be manufactured into lightweight panels with complex geometries while requiring relatively low energy inputs compared with conventional composite manufacturing routes [21,22,24,25,26,27].

In automotive applications, weight reduction remains an important strategy for improving fuel efficiency and reducing lifecycle greenhouse gas emissions, creating opportunities for bio-based materials as replacements for petroleum-derived interior components [12,21,26,27,107]. Mycelium-based panels may therefore be suitable for interior trim systems, acoustic elements, and semi-structural components where low mass, vibration damping, and sustainability are desirable characteristics [21,22,24,25,26,27].

However, current mycelium composites generally exhibit lower strength and stiffness than conventional structural composites because their performance is constrained by porosity, moisture sensitivity, and variability in fungal growth characteristics [21,24,27,28,31]. Consequently, their near-term adoption is expected to focus primarily on non-load-bearing applications, although ongoing efforts involving densification, reinforcement, and chemical modification may progressively expand their structural capabilities.

### 6.4. Functional Materials

Beyond structural and packaging applications, the rich chemistry of fungal cell walls provides opportunities for developing advanced functional materials. Chitin, chitosan, β-glucans, proteins, and associated polysaccharides contain abundant hydroxyl, amino, and acetamide groups that can interact with a wide range of chemical species through hydrogen bonding, electrostatic attraction, and coordination interactions [23,25,33,37,39]. As discussed in Section 5, chemical functionalisation strategies can further tailor these interactions by modifying surface charge, polarity, and accessibility of active binding sites.

One important application area is the adsorption and removal of heavy metals from contaminated water systems. The presence of amino, hydroxyl, and carboxyl-containing functionalities enables fungal biomaterials to bind dissolved metal ions through ion-exchange and complexation mechanisms, making mycelium-derived adsorbents attractive for environmental remediation [25,108,109,110,111]. Similarly, fungal materials have demonstrated the ability to remove diverse organic pollutants and dyes through a combination of adsorption and biodegradation pathways, highlighting their potential in wastewater treatment technologies [23,111,112,113,114].

Recent work has also demonstrated the potential of chemically modified mycelial networks for capturing emerging contaminants such as microplastics, where both surface chemistry and fibrous morphology contribute to adsorption performance. Functionalisation of the mycelium surface can enhance electrostatic interactions and improve capture efficiency relative to untreated materials, illustrating the importance of the modification strategies discussed in Section 5 [23,25,37,39,70].

The highly interconnected pore network produced by hyphal self-assembly further provides opportunities for filtration and separation applications because it combines fluid permeability with large internal surface area and chemically active interfaces [21,22,23,25,26]. Consequently, mycelium-based materials are increasingly being explored as sustainable platforms for adsorbents, filtration media, bioseparation systems, membrane supports, and multifunctional environmental materials.

Advanced MBC materials containing carbon nanotubes have been shown to have great potential due to a broad range of properties, such as self-healing, self-sensing, improved mechanical properties, all while remaining low density. These materials have been proposed for use in smart sensing within human care due to their ability to detect pressure and bending deformation [115]. However, the incorporation of non-biodegradable nanomaterials introduces important sustainability trade-offs. In contrast to purely bio-based MBC systems, CNT-containing composites may exhibit reduced biodegradability and more complex end-of-life management requirements [25,116]. Consequently, while such hybrid materials are promising for high-value functional applications, including sensing and advanced engineering systems, they may be less compatible with circular economy and biodegradation objectives than fully bio-based alternatives. Future research should therefore evaluate both performance gains and lifecycle impact when selecting reinforcement strategies.

## 7. Sustainability and Life-Cycle Considerations

### 7.1. Environmental Impact

The growing interest in MBCs is largely driven by the need to reduce the environmental burdens associated with conventional petroleum-derived polymers and composite materials. As traditional plastics and synthetic foams are produced from fossil resources through energy-intensive manufacturing processes, they are typically associated with substantial greenhouse gas emissions and long-term environmental persistence [1,4,21,24,26]. In contrast, mycelium-based materials are manufactured through biological growth using renewable carbon sources, enabling the partial replacement of fossil-derived feedstocks with biomass-based resources [24,25,26,27,28].

A key sustainability advantage of fungal materials is the relatively low energy requirement of the bio-fabrication process compared with conventional polymer and composite manufacturing. Unlike conventional composite manufacturing methods that frequently rely on elevated temperatures, pressure-assisted consolidation, or chemical polymerisation, fungal cultivation typically occurs under ambient or near-ambient conditions with minimal external energy inputs. Material formation is achieved through the self-assembly of fungal hyphae and enzymatic substrate transformation, reducing the need for energy-intensive processing operations during the primary fabrication stage [21,23,24,26,27,98].

Life-cycle assessments of bio-based materials have consistently demonstrated that the substitution of fossil-derived feedstocks with renewable biomass can reduce carbon emissions and resource depletion when sustainable sourcing and end-of-life management strategies are employed. Furthermore, recent studies have shown that fungal bioprocessing can valorise difficult waste streams, including post-consumer materials, into functional products with insulation and fire-resistant properties, providing additional environmental benefits through waste diversion and resource recovery [6,12,14,23,25,26,27,28,98].

### 7.2. Biodegradation and Chemical Stability

An important characteristic distinguishing MBCs from conventional synthetic materials is their capacity to undergo biological degradation at the end of their service life. As these materials are composed primarily of fungal biomass, cellulose, hemicellulose, lignin, and related biopolymers, they can be metabolised by naturally occurring microorganisms through enzymatic degradation pathways [23,24,25,26,49].

Decomposition occurs through the action of hydrolytic and oxidative enzymes that progressively break down polysaccharides and aromatic biopolymers into smaller molecules that can subsequently enter natural biogeochemical cycles. Cellulases, hemicellulases, and chitin-degrading enzymes contribute to the degradation of carbohydrate-rich components, while oxidative enzymes including laccases and peroxidases facilitate the breakdown of lignin-derived structures and other recalcitrant organic compounds [23,25,33,49,60,61].

The rate and extent of biodegradation are strongly influenced by environmental conditions, material composition, density, moisture availability, and post-processing treatments. Densified or chemically modified materials generally exhibit improved durability and resistance to environmental degradation during service, although these modifications may also slow biodegradation after disposal [21,24,27,28,31].

This balance between service-life stability and eventual biodegradability represents one of the key design considerations for mycelium-based materials, particularly in applications requiring long-term durability while retaining environmentally benign end-of-life behaviour [23,24,25,26,27].

### 7.3. Circular Economy

**Figure 5:**
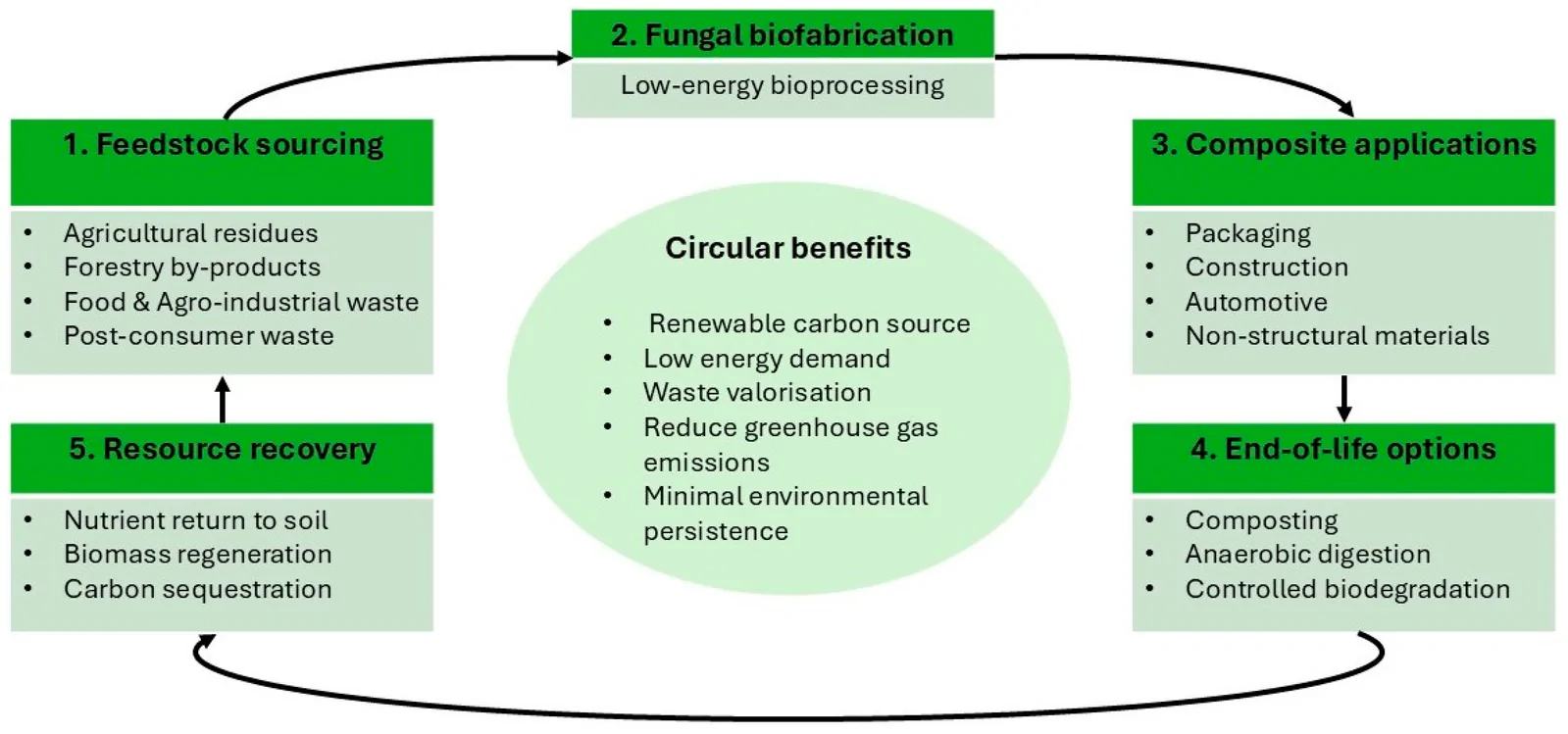
Circular economy framework for MBCs. Renewable and waste-derived feedstocks are converted into functional and environmental remediation materials through fungal bio-fabrication. Products can be applied in packaging, construction, automotive, and functional material applications before entering end-of-life pathways such as composting, biodegradation, or resource recovery, thereby supporting closed-loop and low-carbon material systems.

Mycelium-based materials align closely with circular economy principles because they enable the conversion of agricultural, forestry, industrial, and post-consumer waste streams into value-added products through biological processing. Unlike many conventional manufacturing systems that follow a linear “take-make-dispose” model, fungal biofabrication creates opportunities to retain material value within the economy while reducing waste generation and resource consumption [13,14,23,24,25,26,27,28,98]. Figure 5 summarises the circular economy framework associated with fungal bio-fabrication, highlighting material flows from feedstock sourcing through product application and end-of-life resource recovery.

A major advantage of fungal biotechnology is its ability to utilise a diverse range of lignocellulosic feedstocks, including straw, hemp residues, sawdust, corn stover, paper waste, food-processing by-products, and other biomass resources that are often underutilised or discarded. Through enzymatic transformation and hyphal network formation, fungi convert these heterogeneous resources into cohesive materials with useful structural and functional properties [21,23,24,25,28,31,49,98].

Recent investigations have further expanded this concept by demonstrating fungal growth on complex post-consumer waste streams, including polyurethane-rich mattress waste, resulting in the formation of functional insulation materials with enhanced thermal stability and resource recovery potential [21,23,24,25,28,31,49,98]. These material flows are summarised in Figure 5.

At the end of their service life, mycelium-based products may be composted, biodegraded, or potentially recycled into subsequent biomass utilisation pathways, creating the possibility of closed-loop material systems that minimise environmental burdens while maximising resource efficiency [21,24,25,26,27].

### 7.4. Comparison with Conventional Materials

The suitability of MBCs as alternatives to conventional materials depends on balancing engineering performance with sustainability advantages. A quantitative comparison between MBCs and commonly used synthetic and engineered materials is presented in Table 6.

Table 6 demonstrates that although MBCs generally exhibit lower mechanical strength than structural wood products and fibre-reinforced composites, they can achieve thermal insulation performance comparable to commercial insulation materials while offering superior circularity, renewable feedstock utilisation, and biodegradability.

From a mechanical perspective, MBCs exhibit lower compressive strength than conventional structural materials. However, the wide range of reported values reflects significant differences in processing conditions [21,24,27,28,30,64]. Compressive strengths below approximately 0.05 MPa are generally associated with highly porous, untreated composites produced with minimal densification, whereas values exceeding 4 MPa have typically been achieved through hot-pressing, mechanical densification, optimised substrate selection, and enhanced fungal network consolidation. Densification reduces pore volume, increases contact points between fungal and substrate polymers, and enhances load transfer throughout the composite network, resulting in substantially higher compressive strength and stiffness [94,118]. Consequently, the reported compressive strength range should not be interpreted as representing a single material class but rather a spectrum of engineered materials whose performance is strongly influenced by process–structure–property relationships [21,24,27,28,30,64]. These values are significantly lower than those of structural wood products, which typically exhibit compressive strengths of 20–60 MPa, and far below fibre-reinforced polymer composites, which commonly exceed 100 MPa [12,21,24,27,28,64,107]. Consequently, current mycelium-based materials are best suited to non-load-bearing and semi-structural applications where low density and sustainability are prioritised over maximum mechanical performance.

In contrast, the thermal insulation performance of mycelium composites is highly competitive with conventional insulation materials. As shown in Table 6, the thermal insulation performance of MBCs is highly competitive with conventional insulation materials [21,22,24,27,30]. Notably, Nguyen et al. [98] reported a thermal conductivity of 0.048 ± 0.002 W m^−1^ K^−1^ for mycelium-mattress waste biocomposites, demonstrating performance comparable to commercially available sustainable insulation materials while simultaneously valorising post-consumer waste streams.

Mycelium-based materials also exhibit promising fire-resistant behaviour relative to many petroleum-derived foams. Whereas EPS and polyurethane foams undergo rapid thermal degradation and can release combustible volatile compounds during burning, fungal biomaterials often form protective char layers due to the presence of chitin, chitosan, and other nitrogen-containing biopolymers [21,22,23,24,27,30]. Nguyen et al. [98] demonstrated that chemically treated mycelium-mattress composites retained 85–93 wt.% residual mass at approximately 1000 °C, substantially exceeding the thermal stability of commercial glass wool insulation tested under identical conditions, which retained no measurable mass at 992 °C.

Perhaps the greatest advantage of mycelium-based materials lies in sustainability metrics rather than absolute thermal and mechanical performance. Conventional plastics and synthetic foams are derived primarily from non-renewable fossil resources and frequently persist in the environment for decades or centuries after disposal [1,4,8,21,26]. In contrast, mycelium composites can be fabricated from agricultural residues, forestry by-products, food waste, paper waste, and even post-consumer polymeric waste streams, while retaining the ability to biodegrade under suitable environmental conditions at end-of-life [24,25,26,28,98]. This combination of renewable feedstocks, low-energy biological manufacturing, waste valorisation, and biodegradability distinguishes mycelium-based materials from most conventional engineering materials and supports their integration into circular economy frameworks.

Overall, these comparisons highlight that the principal competitive advantage of MBCs lies not in maximising mechanical or other functional performance, but in achieving an optimal balance between functionality, sustainability, biodegradability, and circular resource utilisation. Continued advances in chemical functionalisation, densification, hybrid reinforcement, and process control are expected to further narrow the performance gap between mycelium-based materials and conventional products, particularly for packaging, insulation, automotive interiors, and other non-load-bearing applications.

## 8. Challenges and Future Perspectives

### 8.1. Current Challenges

Despite significant advances in fungal biomaterials research, several scientific and technological barriers continue to limit the widespread adoption and commercial implementation of MBCs. One of the most persistent challenges is the inherent variability associated with fungal growth processes, which can result in substantial differences in material composition, microstructure, and performance even when similar cultivation conditions are employed. Variations in fungal species, strain selection, substrate chemistry, moisture content, temperature, oxygen availability, and cultivation duration have all been shown to influence hyphal morphology, cell-wall composition, and network architecture, leading to significant fluctuations in density, compressive strength, thermal conductivity, and moisture sensitivity [21,22,24,27,28,30,31].

The magnitude of variability reported for MBCs remains substantially higher than that observed for conventional engineering materials. Compressive strength values range from <0.05 MPa to >4 MPa, representing almost two orders of magnitude variation across studies. Similarly, thermal conductivity ranges from 0.025 to 0.080 W m^−1^ K^−1^ despite comparable material classifications. This variability highlights the current absence of robust process–structure–property relationships and remains one of the principal barriers to industrial-scale deployment and commercialisation [21,22,24,27,28,30,64,98,117].

A second major challenge is the limited molecular-level understanding of the relationships between fungal biosynthesis, substrate transformation, microstructure development, and resulting material properties. Although chitin, chitosan, β-glucans, proteins, and lignocellulosic substrate components are known to contribute to composite performance, the precise mechanisms governing interfacial bonding, stress transfer, moisture transport, and degradation remain incompletely understood. In particular, quantitative process–structure–property relationships comparable to those used in conventional polymer and composite engineering are still largely absent, making predictive material design difficult [21,22,23,24,25,27,28,31,33].

Scale-up and manufacturing reproducibility also remain significant obstacles to commercialisation. Most published studies have been conducted at laboratory scale using small moulds and highly controlled cultivation conditions, whereas industrial production requires reliable control over contamination, oxygen transfer, moisture distribution, growth kinetics, and material uniformity across large volumes. Even relatively minor differences in substrate packing density or local environmental conditions can lead to heterogeneous colonisation and property gradients throughout the final product, complicating quality assurance and certification processes [21,24,26,27,28,30,31].

Additionally, durability-related issues such as moisture sensitivity, biological degradation during service, and long-term dimensional stability continue to restrict the use of mycelium composites in demanding engineering environments [21,24,27,28,98]. Although chemical modification, densification, and hybrid reinforcement strategies have demonstrated promising improvements in mechanical performance, moisture resistance, and environmental durability, achieving a balance between long-term durability and end-of-life biodegradability remains a complex materials-design challenge. The most critical limitations and corresponding future research opportunities are outlined in Table 7.

The challenges identified in Table 7 highlight the multidisciplinary nature of future MBC research and emphasise the need for closer integration between fungal biotechnology, materials chemistry, manufacturing science, computational modelling, and data-driven process optimisation.

### 8.2. Emerging Directions

One of the most promising future research directions involves the application of synthetic biology and metabolic engineering to tailor fungal biosynthetic pathways for advanced material production. Recent developments in fungal genetics, CRISPR-based genome editing, and pathway engineering have created opportunities to manipulate the production of structural biopolymers such as chitin, chitosan, β-glucans, proteins, and extracellular matrix components. By controlling polymer composition, degree of acetylation, branching density, cell-wall architecture, and extracellular matrix production, it may become possible to engineer fungal strains that produce materials with predetermined mechanical, thermal, or chemical properties [21,23,24,25,33,37].

Advanced materials chemistry is expected to play an equally important role in overcoming current performance limitations. As discussed in Section 5, chemical functionalisation, grafting techniques, silane-coupling strategies, enzymatic crosslinking, and hybrid inorganic–organic systems have already demonstrated the ability to improve hydrophobicity, thermal stability, fire resistance, and adsorption performance [23,24,25,26,27,28,31,98]. Future work is likely to focus on developing multifunctional materials that integrate structural performance with sensing, filtration, self-healing, antimicrobial activity, or environmental remediation capabilities.

The integration of fungal materials with additive manufacturing and digital fabrication technologies represents another promising emerging research area. Conventional mycelium fabrication is often constrained by mould geometries and growth-induced variations, whereas additive manufacturing offers opportunities for precise control over architecture, density gradients, and material distribution. Recent studies incorporating wood flour, hydrogels, nanoclays, and fungal growth into three-dimensional printed structures have demonstrated substantial improvements in mechanical performance and geometric complexity compared with conventional growth methods [21,23,25,26,27,28,31].

Particular promise exists in the development of digitally controlled bio-fabrication platforms integrating predictive modelling, automated cultivation control, and advanced manufacturing techniques where fungal growth, substrate composition, environmental conditions, and geometric design are integrated into a single manufacturing system. Such approaches could allow the production of functionally graded materials with spatially tailored properties that are difficult or impossible to achieve using traditional composite processing methods.

### 8.3. Future Vision

The long-term vision for fungal biomaterials extends beyond simply replacing existing synthetic materials. Instead, mycelium-based systems have the potential to establish an entirely new materials paradigm in which biological growth, renewable feedstocks, and advanced materials engineering are integrated into a unified manufacturing platform [21,23,24,25,26].

Future MBCs are expected to become increasingly scalable, reproducible, application-specific, and performance-predictable through improvements in strain engineering, substrate selection, process control, and post-processing technologies [21,23,27,28,31]. The transition from laboratory-scale prototypes to industrially manufactured products will require robust quality-control frameworks, standardised testing protocols, and predictive process–structure–property models comparable to those used in conventional polymer and composite manufacturing.

In terms of performance, future materials will likely combine the sustainability advantages of fungal systems with significantly enhanced mechanical and functional properties. Current mycelium-based insulation materials already achieve thermal conductivities comparable to many commercial insulation products, while recent fungal-mattress-waste composites have shown thermal conductivities of 0.048 ± 0.002 W m^−1^ K^−1^ alongside exceptional thermal stability and fire resistance [21,22,27,30,98]. Continued optimisation through densification, hybrid reinforcement, and molecular engineering is expected to further improve these properties while maintaining low density and renewable content.

Beyond structural and construction applications, future fungal materials may serve as multifunctional platforms capable of combining mechanical support with environmental remediation, filtration, adsorption, sensing, thermal management, and biological responsiveness [23,24,25,26,27]. The convergence of synthetic biology, advanced chemistry, and digital manufacturing therefore has the potential to transform mycelium-based materials from niche sustainable alternatives into a broadly applicable class of high-performance bioengineered composites.

Ultimately, the greatest opportunity for MBCs lies in their ability to couple waste valorisation, low-energy manufacturing, biodegradability, and tuneable functionality within a single material platform. Rather than competing directly with conventional engineering materials across all applications, future MBCs are likely to occupy a unique position where environmental performance and material functionality are simultaneously optimised, supporting the transition toward a circular and bio-based materials economy.

## 9. Conclusions

Fungal biotechnology represents a transformative approach to the development of sustainable biocomposites, enabling the direct conversion of complex polymer substrates into structurally integrated materials. This review highlights that the performance of mycelium-based composites is fundamentally governed by the interplay between fungal biosynthesis, substrate chemistry, and processing strategies. Key biopolymers, including chitin, chitosan, and β-glucans, provide a chemically versatile matrix whose structure and functionality can be tailored through both biological and post-processing interventions.

The chemical evolution of lignocellulosic substrates during fungal colonisation, driven by enzymatic depolymerisation and interfacial bonding, plays a central role in defining composite architecture and properties. Advances in spectroscopic, thermal, and structural characterisation have enabled deeper insights into process–structure–property relationships, particularly in relation to density, porosity, and interpolymer interactions. Processing strategies, especially densification and thermal treatment, further allow the tailoring of mechanical strength, thermal insulation, and durability, demonstrating strong potential for scalable material optimisation.

Applications across packaging, construction, and functional materials underline the versatility of mycelium-based systems, while their biodegradability and compatibility with circular economy principles position them as viable alternatives to conventional composites. However, challenges remain in controlling compositional variability, improving reproducibility, establishing predictive design frameworks, and advancing molecular-level understanding.

Furthermore, continued integration of fungal biotechnology, advanced materials chemistry, digital manufacturing, and predictive modelling will be essential for unlocking the full potential of MBCs as next-generation sustainable engineering materials.

## Figures and Tables

**Table 1:** Major fungal cell-wall components and their contribution to MBC properties [32,33,34].

Component	Molecular Structure	Role in MBCs
Chitin	β-(1 → 4)-N-acetylglucosamine	Reinforcement
Chitosan	Deacetylated chitin	Functionalisation
β-Glucans	β-(1 → 3)/(1 → 6) polysaccharides	Toughness
Proteins	Glycoproteins	Interfacial bonding

**Table 2:** Major fungal enzymes involved in substrate transformation [48,49,56,60,61,62,63,64,65,66].

Enzyme	Target	Function
Endoglucanase	Cellulose	Chain cleavage
β-Glucosidase	Cellobiose	Glucose release
Xylanase	Hemicellulose	Backbone modification
Laccase	Lignin	Oxidative breakdown
MnP	Lignin	Radical generation
LiP	Lignin	Aromatic cleavage

**Table 3:** Relationship between processing parameters and material properties [21,22,24,27,30,64].

Parameter	Effect on Growth	Effect on Properties
Temperature	Growth kinetics	Density
Humidity	Colonisation	Porosity
Oxygen	Biomass formation	Homogeneity
Hot pressing	None	Strength increase
Compression	None	Porosity decrease

**Table 4:** Chemical modification strategies for mycelium materials [22,65,66,67,68,69,70,71,72].

Method	Modification	Main Effect	Performance	Scalability	Industrial Potential
Deacetylation	Chitin to chitosan	Thermal stability	Fire resistance increase	Moderate	Moderate–high
EPTAC grafting	Surface charge modification	Adsorption	~2× increase	Moderate	Moderate
Silanisation	Hydrophobic groups	Wettability	Water resistance increase	Moderate	High
Crosslinking	Network stabilisation	Mechanical performance	Strength increase	Moderate	High
Hybrid additives	Inorganic reinforcement	Thermal/mechanical	Multi-functional	High	High

**Table 5:** Comparison of different MBCs based on the substrates used and compressive strength.

Substrate	Application	Compressive Strength	Reference
Coffee husk	Mycoblock	283 kPa	[84]
Saw dust	Mycoblock	605 kPa	[84]
Bagasse	Mycoblock	559 kPa	[84]
Cotton seed hull	Latex composite	422 kPa	[85]
Sawdust/psyllium husk	Extrusion	N/A (failed)	[86]

**Table 6:** Comparison of MBCs and conventional materials [21,24,27,107,117].

Property	MBC	Expanded Polystyrene (EPS)	Polyurethane (PU) Foam	Medium-Density Fibreboard (MDF)	Glass-Fibre-Reinforced Polymer (GFRP)
Density (kg m^−3^)	50–350	15–350	30–300	600–800	1500–2000
Compressive strength (MPa)	0.02–4.0	0.07–0.70	0.10–2.00	20–60	>100
Thermal conductivity (W m^−1^ K^−1^)	0.025–0.080	0.030–0.040	0.022–0.035	0.10–0.15	0.20–0.40
Renewable	Yes	No	No	Partial	No
Biodegradable	Yes	No	No	Limited	No

**Table 7:** Key challenges and future research opportunities.

Challenge	Current Limitation	Future Research Opportunities
Growth variability	Poor reproducibility	AI-guided cultivation
Moisture sensitivity	Limited durability	Surface modification
Mechanical properties	Low strength	Hybrid reinforcement
Scale-up	Batch inconsistency	Automated bioreactors
Predictive design	Limited modelling	Digital twins
Fire standards	Limited certification	Building-code validation

## Data Availability

No new data were created or analyzed in this study. Data sharing is not applicable to this article.

## Abbreviations

The following abbreviations are used in this manuscript:

MBCs	Mycelium-based biocomposites
DA	Degree of acetylation
EPS	Expanded polystyrene
PU	Polyurethane
MDF	Medium-density fibreboard
GFRP	Glass-fibre-reinforced polymer
EPTAC	2,3-Epoxypropyltrimethylammonium chloride
APTES	Aminopropyl triethoxysilane
PEG	Poly(ethylene glycol)
NaOH	Sodium hydroxide
H_2_O_2_	Hydrogen peroxide
MnP	Manganese peroxidase
LiP	Lignin peroxidase
UDP	Uridine diphosphate
UV	Ultraviolet
CO_2_	Carbon dioxide
OH	Hydroxyl group
NH_2_	Amino group
NHCOCH_3_	Acetamide group
CNT	Carbon nanotube
CRISPR	Clustered regularly interspaced short palindromic repeats
AI	Artificial intelligence
pH	Potential of hydrogen
°C	Degrees Celsius
MPa	Megapascal
wt.%	Weight percent
